# New refinement of the crystal structure of Zn(NH_3_)_2_Cl_2_ at 100 K

**DOI:** 10.1107/S2056989019011757

**Published:** 2019-08-30

**Authors:** Trpimir Ivšić, David Wenhua Bi, Arnaud Magrez

**Affiliations:** a Laboratory of Physics of Complex Matter, Ecole Polytechnique Fédérale de Lausanne, Switzerland; b Crystal Growth Facility, Ecole Polytechnique Fédérale de Lausanne, Switzerland

**Keywords:** crystal structure, redetermination, low temperature, high resolution data set

## Abstract

The crystal structure of [ZnCl_2_(NH_3_)_2_], diamminedi­chlorido­zinc, was re-investigated at low temperature, revealing the positions of the hydrogen atoms and thus a deeper insight into the hydrogen-bonding scheme in the crystal packing. In comparison with previous crystal structure determinations [MacGillavry & Bijvoet (1936 ▸). *Z. Kristallogr.* **94**, 249–255; Yamaguchi & Lindqvist (1981 ▸). *Acta Chem. Scand.* **35**, 727–728], an improved precision of the structural parameters was achieved. In the crystal, tetra­hedral [Zn(NH_3_)_2_Cl_2_] units (point-group symmetry *mm*2) are linked through N—H⋯Cl hydrogen bonds into a three-dimensional network.

## Abstract 2

The crystal structure of Zn(NH_3_)_2_Cl_2_ was redetermined at low temperature, revealing the positions of the hydrogen atoms.

## Chemical context   

Zn(NH_3_)_2_Cl_2_ is found in discharged zinc–air batteries. It is formed by dissolution of the zinc electrode in an ZnCl_2_–NH_4_Cl electrolyte. At high Zn^2+^ concentrations, the cations subsequently undergo complex formation with NH_3_ and Cl^−^ (Clark *et al.*, 2017 ▸). Moreover, in the last century, Zn(NH_3_)_*n*_Cl_2_ phases with *n* = 0.167–6 have been intensively investigated since these materials appear to be by-products of hydro­cracking of heavy-oil fractions. The monoammine salt with *n* = 1 and the diammine salt with *n* = 2 are the most studied members in this family. Thermal stability, thermomechanical and thermogravimetric behaviour are well documented for these systems (Gardner *et al.*, 1989 ▸).

The crystal-structure model of Zn(NH_3_)_2_Cl_2_ was to this day incomplete, lacking the positions of hydrogen atoms. The crystal structure of Zn(NH_3_)_2_Cl_2_ was originally determined by MacGillavry & Bijvoet (1936 ▸) without refinement of atomic positions [COD number ID 1010197; ICSD number 26136; PDF4 number 01-074-0506]. This incompleteness was rather normal considering the X-ray techniques and measurement conditions at that time. An improved structure model was obtained decades later from a room-temperature data set collected on a four-circle diffractometer by Yamaguchi & Lindqvist (1981 ▸), however without hydrogen-atom positions [PDF4 number 04-015-4717]*.* One more record of this phase in PDF4 [number 00-024-1435] can be found, however with a very limited X-ray powder diffraction pattern range from 5–63°/2*θ*, which is unsuitable for Rietveld refinement.

Large single crystals of Zn(NH_3_)_2_Cl_2_ were obtained as a side product of a chemical vapour transport (CVT) reaction, which allowed the reinvestigation of the crystal structure at low temperature with a particular focus on determination of the H-atom positions.

## Structural commentary   

**Figure 1:**
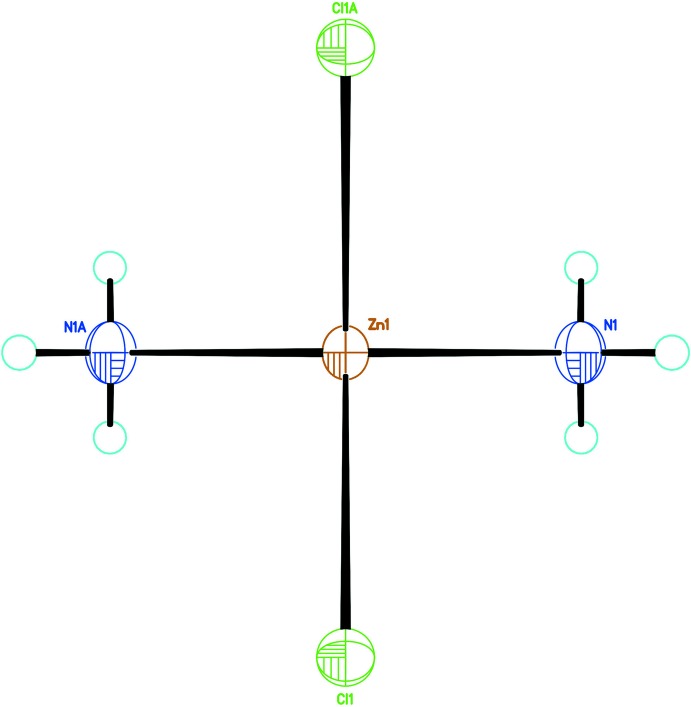
The tetra­hedral [Zn(NH_3_)_2_Cl_2_] group. H atoms (cyan circles) are plotted with arbitrary size. [Symmetry code: (A) 1 − *x*, 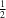 − *y*, *z*.].

**Figure 2:**
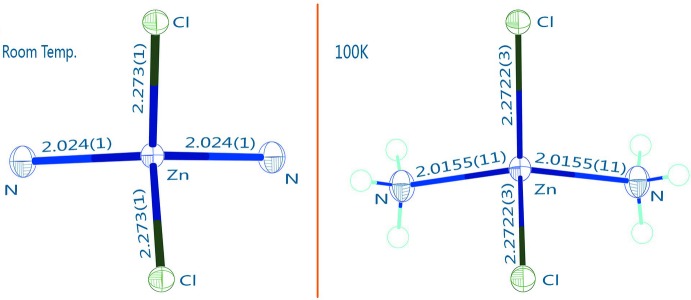
Comparison of the [ZnN_2_Cl_2_] tetra­hedra from the room temperature data (left; Yamaguchi & Lindqvist, 1981 ▸) and the present 100 K data (right). Bond lengths for the room-temperature data were calculated with *DIAMOND* (Brandenburg, 2006 ▸).

**Figure 3:**
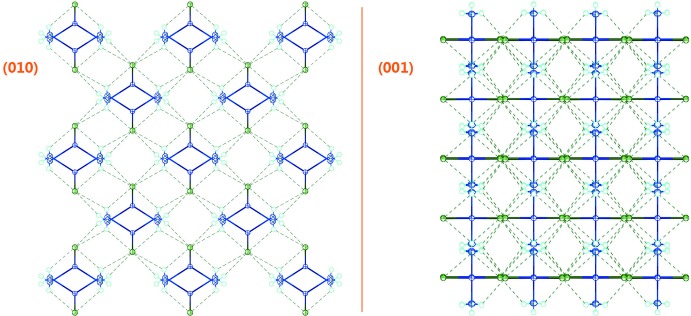
Packing view along the *b* axis (left) and the *c* axis (right), showing N—H⋯Cl inter­actions between adjacent [Zn(NH_3_)_2_Cl_2_] tetra­hedra.

As shown in Fig. 1 ▸, the new structure refinement of Zn(NH_3_)_2_Cl_2_ (100 K data) confirms the former model by Yamaguchi & Lindqvist (1981 ▸). The small shrinkage of the unit cell at low temperature is mainly due to lattice dynamics. However, the bond lengths within the Zn(NH_3_)_2_Cl_2_ tetra­hedron refined from 100 K data are similar to the ones obtained from room-temperature data, but with higher precision. This confirms that the tetra­hedron is a very rigid building block of the structure (Fig. 2 ▸). Two independent H atoms, one in a general position (H1*A*) and one on a mirror plane (H1*B*), bond to the nitro­gen atom with refined bond lengths of N1—H1*A* = 0.825 (17) Å and N1—H1*B* = 0.74 (4) Å, respectively. Hydrogen-bonding inter­actions of weak-to-medium strengths (Table 1 ▸) between the NH_3_ group and the Cl ligands of adjacent tetra­hedra lead to formation of a three-dimensional network, as shown in Fig. 3 ▸.

## Synthesis and crystallization   

Zn(NH_3_)_2_Cl_2_ was obtained during the course of a chemical vapour transport experiment. Prior to use, a silica tube sealed at one end (Ø = 15 mm, wall thickness = 2 mm) was heated under vacuum to 1073 K overnight. Zinc oxide (317 mg) and previously dried ammonium chloride (190 mg) were pressed into pellets and put into the pretreated silica tube. After evacuation to 10^−6^ mbar, the tube was sealed and placed into a horizontal tube furnace. The temperature at the source was slowly heated to 1173 K and maintained at this value for the whole duration of growth. At the low-temperature zone (873 K), a colourless powder mixed with high-quality single crystals of Zn(NH_3_)_2_Cl_2_ with an irregular morphology was collected after complete cooling to room temperature. Since the thermal stability of Zn(NH_3_)_2_Cl_2_ is limited as it already decomposes at 463 K (Gardner *et al.*, 1989 ▸), it is very likely that Zn(NH_3_)_2_Cl_2_ crystallized at the very end of the cooling process.

## Refinement   

Crystal data, data collection and structure refinement details are summarized in Table 2 ▸. Unlike the two previous refinements in space group *Imam*, the unit cell was chosen to be in the standard setting (*Imma*) for space group No. 74. Three reflections (020, 002, 004) affected by the incident beam-stop were omitted. The two H atoms were refined freely.

## Supplementary Material

Crystal structure: contains datablock(s) I. DOI: 10.1107/S2056989019011757/wm5517sup1.cif

Structure factors: contains datablock(s) I. DOI: 10.1107/S2056989019011757/wm5517Isup2.hkl

CCDC reference: 1949371

Additional supporting information:  crystallographic information; 3D view; checkCIF report

## Figures and Tables

**Table 1:** Hydrogen-bond geometry (Å, °)

*D*—H⋯*A*	*D*—H	H⋯*A*	*D*⋯*A*	*D*—H⋯*A*
N1—H1*A*⋯Cl1^i^	0.825 (17)	2.806 (18)	3.4552 (9)	137.0 (17)
N1—H1*A*⋯Cl1^ii^	0.825 (17)	2.897 (18)	3.5505 (9)	137.6 (16)
N1—H1*B*⋯Cl1^iii^	0.74 (4)	2.95 (3)	3.5556 (10)	141 (1)
N1—H1*B*⋯Cl1^iv^	0.74 (4)	2.95 (3)	3.5556 (10)	141 (1)

Symmetry codes: (i) 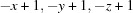; (ii) 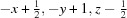; (iii) 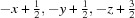; (iv) 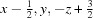.

**Table 2:** Experimental details

Crystal data	
Chemical formula	[ZnCl_2_(NH_3_)_2_]
*M*_r_	170.34
Crystal system, space group	Orthorhombic, *I* *m* *m* *a*
Temperature (K)	100
*a*, *b*, *c* (Å)	7.7077 (2), 8.0226 (2), 8.4526 (3)
*V* (Å^3^)	522.67 (3)
*Z*	4
Radiation type	Mo *K*α
μ (mm^−1^)	5.56
Crystal size (mm)	0.26 × 0.18 × 0.16

Data collection	
Diffractometer	Rigaku SuperNOVA
Absorption correction	Analytical (*CrysAlis PRO*; Rigaku OD, 2019 ▸)
*T*_min_, *T*_max_	0.277, 0.454
No. of measured, independent and observed [*I* > 2σ(*I*)] reflections	2303, 708, 678
*R*_int_	0.017
(sin θ/λ)_max_ (Å^−1^)	0.833

Refinement	
*R*[*F* ^2^ > 2σ(*F* ^2^)], *wR*(*F* ^2^), *S*	0.015, 0.036, 1.04
No. of reflections	708
No. of parameters	25
H-atom treatment	All H-atom parameters refined
Δρ_max_, Δρ_min_ (e Å^−3^)	0.50, −0.55

Computer programs: *CrysAlis PRO* (Rigaku OD, 2019 ▸), *SHELXT* (Sheldrick, 2015*a* ▸), *SHELXL2018* (Sheldrick, 2015*b* ▸), *DIAMOND* (Brandenburg, 2006 ▸) and *publCIF* (Westrip, 2010 ▸).

## supplementary crystallographic information

### Crystal data

**Table:**

[ZnCl_2_(NH_3_)_2_]	*D*_x_ = 2.165 Mg m^−^^3^
*M**_r_* = 170.34	Mo *K*α radiation, λ = 0.71073 Å
Orthorhombic, *I**m**m**a*	Cell parameters from 1597 reflections
*a* = 7.7077 (2) Å	θ = 3.6–37.4°
*b* = 8.0226 (2) Å	µ = 5.56 mm^−^^1^
*c* = 8.4526 (3) Å	*T* = 100 K
*V* = 522.67 (3) Å^3^	Irregular, colourless
*Z* = 4	0.26 × 0.18 × 0.16 mm
*F*(000) = 336	

### Data collection

**Table:**

Rigaku SuperNOVA diffractometer	678 reflections with *I* > 2σ(*I*)
Radiation source: micro-focus sealed X-ray tube	*R*_int_ = 0.017
ω scans	θ_max_ = 36.3°, θ_min_ = 3.5°
Absorption correction: analytical (CrysAlis PRO; Rigaku OD, 2019)	*h* = −12→8
*T*_min_ = 0.277, *T*_max_ = 0.454	*k* = −13→12
2303 measured reflections	*l* = −12→14
708 independent reflections	

### Refinement

**Table:**

Refinement on *F*^2^	Hydrogen site location: difference Fourier map
Least-squares matrix: full	All H-atom parameters refined
*R*[*F*^2^ > 2σ(*F*^2^)] = 0.015	*w* = 1/[σ^2^(*F*_o_^2^) + (0.0188*P*)^2^] where *P* = (*F*_o_^2^ + 2*F*_c_^2^)/3
*wR*(*F*^2^) = 0.036	(Δ/σ)_max_ = 0.001
*S* = 1.04	Δρ_max_ = 0.50 e Å^−^^3^
708 reflections	Δρ_min_ = −0.55 e Å^−^^3^
25 parameters	Extinction correction: SHELXL2018 (Sheldrick, 2015b), Fc^*^=kFc[1+0.001xFc^2^λ^3^/sin(2θ)]^-1/4^
0 restraints	Extinction coefficient: 0.0043 (6)
Primary atom site location: structure-invariant direct methods	

### Special details

**Table:**

Geometry. All esds (except the esd in the dihedral angle between two l.s. planes) are estimated using the full covariance matrix. The cell esds are taken into account individually in the estimation of esds in distances, angles and torsion angles; correlations between esds in cell parameters are only used when they are defined by crystal symmetry. An approximate (isotropic) treatment of cell esds is used for estimating esds involving l.s. planes.

### Fractional atomic coordinates and isotropic or equivalent isotropic displacement parameters (Å^2^)

**Table:**

	*x*	*y*	*z*	*U*_iso_*/*U*_eq_	
Zn1	0.500000	0.250000	0.61168 (2)	0.01163 (6)	
Cl1	0.500000	0.47954 (3)	0.76915 (3)	0.01375 (6)	
N1	0.28237 (15)	0.250000	0.47949 (13)	0.01603 (17)	
H1A	0.274 (3)	0.3281 (19)	0.416 (2)	0.045 (4)*	
H1B	0.206 (5)	0.250000	0.533 (4)	0.090 (11)*	

### Atomic displacement parameters (Å^2^)

**Table:**

	*U*^11^	*U*^22^	*U*^33^	*U*^12^	*U*^13^	*U*^23^
Zn1	0.01002 (9)	0.01352 (9)	0.01134 (9)	0.000	0.000	0.000
Cl1	0.01288 (11)	0.01346 (11)	0.01490 (11)	0.000	0.000	−0.00253 (7)
N1	0.0136 (4)	0.0211 (4)	0.0134 (4)	0.000	−0.0016 (3)	0.000

### Geometric parameters (Å, º)

**Table:**

Zn1—Cl1^i^	2.2722 (3)	N1—H1A^ii^	0.825 (17)
Zn1—Cl1	2.2722 (3)	N1—H1A	0.825 (17)
Zn1—N1^i^	2.0155 (11)	N1—H1B	0.74 (4)
Zn1—N1	2.0155 (11)		
			
Cl1^i^—Zn1—Cl1	108.282 (14)	Zn1—N1—H1A^ii^	115.0 (13)
N1—Zn1—Cl1	108.951 (17)	Zn1—N1—H1A	115.0 (13)
N1^i^—Zn1—Cl1^i^	108.951 (17)	Zn1—N1—H1B	109 (3)
N1—Zn1—Cl1^i^	108.951 (17)	H1A—N1—H1A^ii^	99 (2)
N1^i^—Zn1—Cl1	108.950 (17)	H1A—N1—H1B	109 (2)
N1^i^—Zn1—N1	112.66 (7)	H1B—N1—H1A^ii^	109 (2)

Symmetry codes: (i) −*x*+1, −*y*+1/2, *z*; (ii) *x*, −*y*+1/2, *z*.

### Hydrogen-bond geometry (Å, º)

**Table:**

*D*—H···*A*	*D*—H	H···*A*	*D*···*A*	*D*—H···*A*
N1—H1*A*···Cl1^iii^	0.825 (17)	2.806 (18)	3.4552 (9)	137.0 (17)
N1—H1*A*···Cl1^iv^	0.825 (17)	2.897 (18)	3.5505 (9)	137.6 (16)
N1—H1*B*···Cl1^v^	0.74 (4)	2.95 (3)	3.5556 (10)	141 (1)
N1—H1*B*···Cl1^vi^	0.74 (4)	2.95 (3)	3.5556 (10)	141 (1)

Symmetry codes: (iii) −*x*+1, −*y*+1, −*z*+1; (iv) −*x*+1/2, −*y*+1, *z*−1/2; (v) −*x*+1/2, −*y*+1/2, −*z*+3/2; (vi) *x*−1/2, *y*, −*z*+3/2.
